# A randomised controlled trial of long NY-ESO-1 peptide-pulsed autologous dendritic cells with or without alpha-galactosylceramide in high-risk melanoma

**DOI:** 10.1007/s00262-023-03400-y

**Published:** 2023-03-07

**Authors:** Nathaniel Dasyam, Katrina J. Sharples, Catherine Barrow, Ying Huang, Evelyn Bauer, Brigitta Mester, Catherine E. Wood, Astrid Authier-Hall, Marina Dzhelali, Tess Ostapowicz, Rajiv Kumar, Jessica Lowe, Alice Maxwell, Olivia K. Burn, Geoffrey M. Williams, Sarah E. Carley, Graham Caygill, Jeremy Jones, Susanna T. S. Chan, Victoria A. Hinder, Jerome Macapagal, Monica McCusker, Robert Weinkove, Margaret A. Brimble, Gavin F. Painter, Michael P. Findlay, P. Rod Dunbar, Olivier Gasser, Ian F. Hermans

**Affiliations:** 1 Malaghan Institute of Medical Research, PO Box 7060, Wellington, 6242 New Zealand; 2 Dunedin School of Medicine, University of Otago, PO Box 56, Dunedin, 9054 New Zealand; 3 Cancer Trials New Zealand, University of Auckland, Private Bag 92019, Auckland, 1142 New Zealand; 4 Capital and Coast District Health Board, Private Bag 7902, Wellington, 6242 New Zealand; 5 School of Chemical Sciences, University of Auckland, PO Box 92019, Auckland, New Zealand; 6 Maurice Wilkins Centre for Molecular Biodiscovery, Private Bag 92019, Auckland, 1142 New Zealand; 7 GlycoSyn, PO Box 31 310, Lower Hutt, 5040 New Zealand; 8 The Ferrier Research Institute, Victoria University of Wellington, PO Box 33436, Lower Hutt, 5046 New Zealand; 9 School of Biological Sciences, University of Auckland, PO Box 92019, Auckland, New Zealand

**Keywords:** Melanoma, Dendritic cell, NKT cell, *α*-Galactosylceramide, NY-ESO-1, Vaccination

## Abstract

**Aim:**

We have previously reported that polyfunctional T cell responses can be induced to the cancer testis antigen NY-ESO-1 in melanoma patients injected with mature autologous monocyte-derived dendritic cells (DCs) loaded with long NY-ESO-1-derived peptides together with *α*-galactosylceramide (*α*-GalCer), an agonist for type 1 Natural Killer T (NKT) cells.

**Objective:**

To assess whether inclusion of *α*-GalCer in autologous NY-ESO-1 long peptide-pulsed DC vaccines (DCV + *α*-GalCer) improves T cell responses when compared to peptide-pulsed DC vaccines without *α*-GalCer (DCV).

**Design, setting and participants:**

Single-centre blinded randomised controlled trial in patients ≥ 18 years old with histologically confirmed, fully resected stage II–IV malignant cutaneous melanoma, conducted between July 2015 and June 2018 at the Wellington Blood and Cancer Centre of the Capital and Coast District Health Board.

**Interventions:**

*Stage I*. Patients were randomised to two cycles of DCV or DCV + *α*-GalCer (intravenous dose of 10 × 10^6^ cells, interval of 28 days). *Stage II.* Patients assigned to DCV + *α*-GalCer were randomised to two further cycles of DCV + *α*-GalCer or observation, while patients initially assigned to DCV crossed over to two cycles of DCV + *α*-GalCer.

**Outcome measures:**

*Primary:* Area under the curve (AUC) of mean NY-ESO-1-specific T cell count detected by ex vivo IFN-γ ELISpot in pre- and post-treatment blood samples, compared between treatment arms at Stage I. *Secondary:* Proportion of responders in each arm at Stage I; NKT cell count in each arm at Stage I; serum cytokine levels at Stage I; adverse events Stage I; T cell count for DCV + *α*-GalCer versus observation at Stage II, T cell count before versus after cross-over.

**Results:**

Thirty-eight patients gave written informed consent; 5 were excluded before randomisation due to progressive disease or incomplete leukapheresis, 17 were assigned to DCV, and 16 to DCV + *α*-GalCer. The vaccines were well tolerated and associated with increases in mean total T cell count, predominantly CD4^+^ T cells, but the difference between the treatment arms was not statistically significant (difference − 6.85, 95% confidence interval, − 21.65 to 7.92; *P* = 0.36). No significant improvements in T cell response were associated with DCV + *α*-GalCer with increased dosing, or in the cross-over. However, the NKT cell response to *α*-GalCer-loaded vaccines was limited compared to previous studies, with mean circulating NKT cell levels not significantly increased in the DCV + *α*-GalCer arm and no significant differences in cytokine response between the treatment arms.

**Conclusions:**

A high population coverage of NY-ESO-1-specific T cell responses was achieved with a good safety profile, but we failed to demonstrate that loading with *α*-GalCer provided an additional advantage to the T cell response with this cellular vaccine design. Clinical trial registration: ACTRN12612001101875. Funded by the Health Research Council of New Zealand.

**Supplementary Information:**

The online version contains supplementary material available at 10.1007/s00262-023-03400-y.

## Introduction

Surgical treatment is effective for early-stage melanoma, but patients with resected advanced disease have a high risk of relapse. Recent clinical evaluation of immune checkpoint blockade in this adjuvant setting has shown a significant reduction of the relative risk of death [1–4], highlighting the role of the immune system in preventing relapse. Additionally, the combination of dabrafenib with trametinib (BRAF/MEK inhibitors) has shown longer duration of survival without relapse or distant metastasis in patients with BRAF V600 mutations [5]. The concept of using safe, targeted vaccines to induce T cell responses with high specificity for defined tumour-antigens remains appealing in this clinical setting, but issues remain about the levels of immunogenicity that can be achieved with current vaccine strategies. Peptide-based vaccination has the advantage that responses can be focussed on the most basic antigenic units—the peptides from tumour-associated antigens presented via major histocompatibility complex (MHC) molecules (human leukocyte antigen (HLA) in humans) to T cells [6, 7]—and not on the vaccine vector itself. However, HLA is highly polymorphic in the human population, so achieving peptide presentation with good population coverage is a significant issue. This can be overcome by the use of long peptides covering a range of different epitopes, assuming information on epitopes presented in the population is known [8, 9]. For some of the more widely studied tumour antigens, such as the cancer testis antigen NY-ESO-1, there are accumulating data on immunogenicity with respect to HLA expression [9–11], making design of long peptides possible.

We recently tested the feasibility and safety of dendritic cell (DC)-based vaccines in high-risk melanoma patients, where the cells were loaded with long NY-ESO-1 peptides designed to give good population coverage on the diverse HLA polymorphism of the human population [12]. Circulating T cells were observed in response to the vaccine in 7 of the 8 patients treated. A major consideration in the design of the vaccine was the inclusion of *α*-galactosylceramide (*α*-GalCer) to stimulate type 1 Natural Killer T cells (NKT cells) [13, 14], as preclinical studies had shown that these innate-like T cells can provide a ready source of stimulatory signals to help priming of conventional T cells [15–18]. With no issues regarding safety of the *α*-GalCer-loaded DC-based vaccines observed, here we treated a larger cohort of patients to specifically evaluate the impact of stimulating NKT cells on the T cell response. The primary objective was to assess whether adding *α*-GalCer to DC-based vaccines would improve T cell responses to peptide antigens in the vaccine (over two vaccination cycles) relative to the DC-based vaccine alone. The secondary objectives were to: (i) assess safety of vaccines incorporating *α*-GalCer, (ii) assess the capacity of vaccines incorporating *α*-GalCer to induce activation and clonal expansion of NKT cells, (iii) assess the capacity of vaccines incorporating *α*-GalCer to boost T cell responses through repeated vaccination, and (iv) assess T cell responses to DC-based vaccines with and without addition of *α*-GalCer in a cross-over setting.

## Materials and methods

### Trial design

**Fig. 1:**
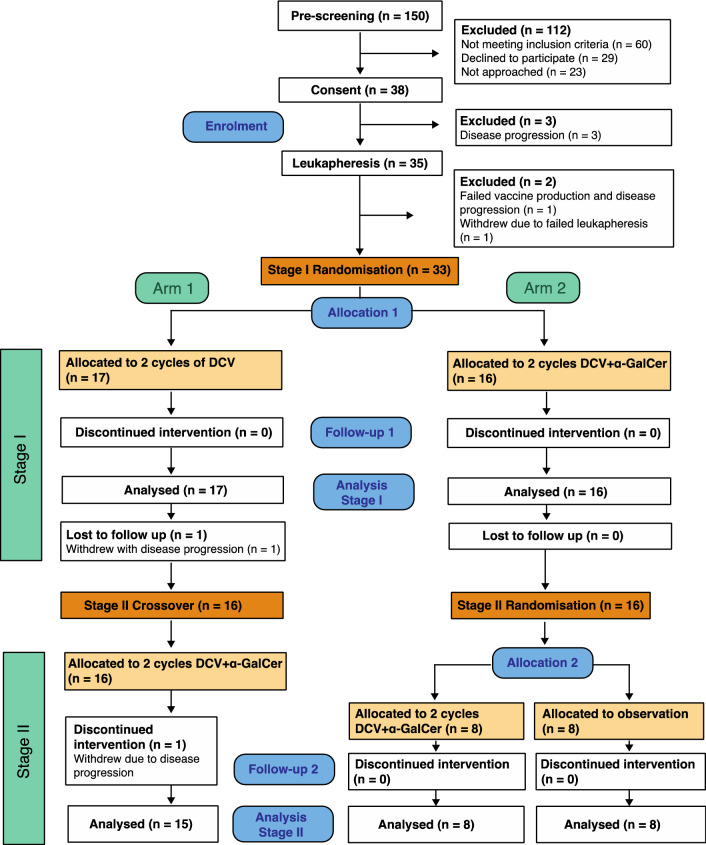
Consort flow diagram. Numbers of patients allocated through pre-screening, consent, leukapheresis and Stage I and II of the study. In Stage I, patients were randomised to receive two cycles of DCV (Arm 1) or two cycles of DCV + *α*-GalCer (Arm 2). In Stage II, patients in Arm 1 then crossed over to receive two cycles of DCV + *α*-GalCer, while patients in Arm 2 were randomised again to receive either two further cycles of DCV + *α*-GalCer or were observed

This was a two-stage study (Fig. 1). In Stage I, patients were randomised 1:1 to receive either two cycles of mature autologous monocyte-derived DCs (MoDCs) loaded with peptides alone (DCV) (Arm 1), or two cycles of mature DCs loaded with peptides plus *α*-GalCer (DCV + *α*-GalCer) (Arm 2), provided as identical vials. The primary and first two secondary objectives were addressed by Stage I. In Stage II, addressing secondary objectives iii and iv, all patients in the DCV arm crossed over to receive two cycles of DCV + *α*-GalCer, while patients in the DCV + *α*-GalCer arm were randomised 1:1 to receive either two further cycles of open-label DCV + *α*-GalCer, or to a control observation only arm. The study, conducted between July 2015 and June 2018, was approved by the Northern B Health and Disability Ethics Committee (ref 13/NTB/5), registered with the Australian and New Zealand Clinical Trials Registry (ACTRN12612001101875) and monitored by an independent Data Monitoring Committee appointed by the Health Research Council of New Zealand.

### Patient population

Patients with histologically confirmed, fully resected American Joint Committee on Cancer (AJCC) Stage II, III or IV malignant cutaneous melanoma who were: ≥ 18 years old; no more than 12 months post-surgery; at least two weeks since most recent surgery; Eastern Cooperative Oncology Group (ECOG) performance status of 0–2; and had normal full blood counts and renal and liver function biochemistry. Patients were excluded if they had: mucosal or ocular melanoma; received prior chemotherapy or radiotherapy within 6 weeks of recruitment; received prior immunotherapy, been diagnosed with another malignancy in the past 3 years (except non-melanoma skin cancer or in situ cancer of the cervix); active infection with Hepatitis B, C or Human immunodeficiency virus (HIV); uncontrolled or unstable autoimmune disease; previous use of long-term immunosuppressive therapy before leukapheresis; co-morbid conditions that would require long-term use (> 1 month) of systemic corticosteroids during study treatment; concurrent major organ dysfunction or other unstable medical condition; or were pregnant or breastfeeding. Patients were recruited by the Wellington Blood and Cancer Centre (WBCC) or Auckland City Hospital.

Eligible patients who gave written informed consent underwent leukapheresis to provide peripheral blood mononuclear cells (PBMCs) from which monocyte-derived DCs were generated, with a maximum of 12 weeks to prepare the vaccine and start study treatment. Both DCV and DCV + *α*-GalCer vaccines were prepared for each patient. Following successful leukapheresis, vaccine production and baseline assessment, patients who still met eligibility criteria were randomised 1:1 to either DCV or DCV + *α*-GalCer using the Cancer Trials New Zealand (CTNZ) central service. The computer-generated random allocation sequence was blocked over time using random block sizes. Only the statistician and database manager had access to randomisation lists. The manufacturing team distributed the appropriate vaccine as randomised but had no other contact with the trial. Participants, research nurses and clinicians were all blinded to the treatment allocation at Stage I.

All clinical procedures and assessments were carried out at WBCC, except for day 1 and day 15 blood draws for Auckland-based patients. Clinical assessments were undertaken at screening and with each vaccine visit until the end of study (28 days after the last vaccination). Adverse events were recorded from the date of consent until end of the study treatment and coded according to Common Terminology Criteria for Adverse Events version 4.0 (CTCAE v4.0). For analysis of T cells and NKT cells, blood was collected 1–6 days before treatment was initiated, immediately before each vaccination, 24 h after vaccination, and 2 and 4 weeks after vaccination. Blood was collected for analysis of serum cytokines 2 days before treatment was initiated, immediately before each vaccination, and 6 h and 24 h after vaccination. Blood samples were enriched for PBMCs within 6 h of collection and immediately cryopreserved for later analysis. Methodology for vaccine manufacture and immune analysis are provided in Supplementary Materials and Methods.

### Treatment

Each cycle of vaccination consisted of a single intravenous dose of 10 × 10^6^ autologous DCs, with an interval of 28 days ± 48 h.

### Outcome measures

The primary outcome measure was the area under the curve (AUC) of the total count of NY-ESO-1_79–116_ and NY-ESO-1_118–143_-specific T cells (measured by ex vivo IFN-γ ELISpot) in baseline blood samples obtained pre-randomisation, and from day 1 pre-treatment, day 2, day 15 and day 29 of cycles 1 and 2 for patients in both treatment arms. There were three replicates for each peptide and five for the negative control (with the same negative control used for each peptide).

The protocol-specified secondary efficacy outcome measures were: (i) NY-ESO-1-specific T cell response over the first two cycles (Yes/No) as measured by ICS of PBMCs following in vitro restimulation on peptide for 10 days, (ii) CD4^+^ or CD8^+^ T cell response (Yes/No) over the first two cycles as measured using ICS following in vitro restimulation, (iii) counts of NKT cells enumerated by flow cytometry of PBMCs using multimeric CD1d/*α*-GalCer complexes at day 1 pre-treatment and day 15 and day 29 of the first two cycles, (iv) counts of NKT cells measured by IFN-γ ELISpot using *α*-GalCer as recall agent, (v) cytokine levels in serum measured using multiplex immunoassays or ELISA for 11 cytokines specified a priori based on earlier studies [19–22] (IL-4, IL-6, IL-10, TNF, IL-12p70, IFN-γ, MCP-1, MIP-1α, MIP-1β, RANTES and IP-10). The specified safety outcome was adverse events from randomisation to end of cycle 2 as defined by the CTCAE version 4.0.

## Statistical analysis

### Primary analysis

The primary analysis compared the AUC across the first two cycles of the log_10_-transformed total T cell counts (measured by IFN-γ ELISpot) for DCV and DCV + *α*-GalCer arms. A linear mixed model with a treatment × time interaction and a spatial power covariance structure was used to estimate the mean overall T cell count at each time point, adjusting for the negative controls. This was a change from the protocol specified analysis caused by some high counts in the negative controls, meaning we could take logs after subtracting the negative control count. Both the baseline and the pre-treatment measures were used for the baseline count. (Note: one patient with inadequate blood samples provided only 2 of 3 replicate cell counts for the peptide measures and 3 of 5 for the negative control). An estimate statement following the fit of the linear model was used to compare the AUCs with a significance level of 0.025 (one-sided). The estimate and 95% confidence interval were translated back to the original scale for ease of interpretation. Sensitivity analyses were planned to adjust for baseline imbalance and missing data.

#### Secondary analyses

For secondary analyses comparing T cell responses measured by ICS, the outcome for each patient was classified as to whether IFN-γ-producing T cells were detected over the first two cycles. Responses were determined using the distribution free sampling approach developed by Moodie et al. [23]. For each patient and each time point, the ICS provided peptide-specific T cell counts (6 replicates for baseline and 3 replicates for later time points). At each time point, we tested a null hypothesis: that the mean at the time point is statistically significantly higher than baseline using a bootstrap test; the comparison across all Stage I time points in each patient was adjusted for multiple comparisons. The difference in proportions with a response in DCV and DCV + *α*-GalCer arms, 95% confidence interval and *p*-value (Fisher’s exact) were calculated. The responses were further categorised as no response, CD4^+^, CD8^+^ or both, and response patterns were compared across arms using a Fisher’s exact test. For the comparison of NKT cell counts measured by flow cytometry, a negative binominal regression was used to compare AUC of the counts of NKT cells in the blood per 100 total CD3^+^ cells across the first two cycles. The comparison of NKT cells measured by IFN-γ ELISpot and the cytokines used the same approach as for the primary outcome. Βackground levels were subtracted (with negative values assigned zero) and then the linear mixed model used without adjustment for the background. For analysis of serum cytokines, the planned analysis compared the AUC across the two cycles of the log_10_-transformed cytokine levels in the DCV and DCV + *α*-GalCer arms. A linear mixed model with a treatment × time interaction and a spatial power covariance structure was applied to each cytokine. The random effect terms were specified to account for the batch variance.

For the Stage II analyses of objective (iii), the outcomes over cycles 3 and 4 in the Stage I DCV + *α*-GalCer group were compared in the group randomised to two further cycles of DCV + *α*-GalCer versus observation alone using the same methods as for the Stage I analyses. For objective iv), outcomes in cycles 1 and 2 (DCV alone) were compared to those in cycles 3 and 4 (DCV + *α*-GalCer) using the same methods as for the Stage I analyses, but including a random effect for individual to provide a within-person comparison.

#### Sample size justification

Our murine studies suggested a four- to fivefold increase in T cell response using vaccines with the addition of *α*-GalCer [24]. A study with 20 patients per group would have 80% power to detect a threefold increase in mean T cell count at the 0.05 level, with a coefficient of variation of 3 [25].

#### Interim analyses

Formal interim analyses were carried out at two time points, the first when 6 patients had completed two cycles to determine final dose (a threefold increase on safe dose established in our earlier dose escalation study), and the second when 20 patients had completed 2 cycles to consider safety and futility.

## Results

### Stage I

#### Patient characteristics and treatment

A summary of the allocation of all enrolled patients is shown in Fig. 1. Of the 38 patients who met eligibility criteria and gave written informed consent, five were excluded before randomisation (progressive disease, *n* = 4; incomplete leukapheresis, *n* = 1). The remaining 33 patients were allocated to receive either two cycles of DCV (Arm 1, *n* = 17) or two cycles of DCV + *α*-GalCer (Arm 2, *n* = 16). Patient demographics and medical history are given in Table 1. Compared to DCV + α-GalCer arm, patients in the DCV arm were older, the time since they were determined disease-free was slightly longer, and greater proportions were male and had received prior radiotherapy. No patients had a history of an auto-immune or inflammatory disorder. Baseline haematology, biochemistry and coagulation parameters were very similar for each arm (Supplementary Table 1).

**Table 1:** Baseline demographics and medical history by treatment group (Stage I)

	Treatment					
	DCV (*n* = 17)	DCV + α-GalCer (*n* = 16)	All (*n* = 33)			
Age at screening						
Median	65.1	58.8	59.5			
Min	33.4	33.5	33.4			
Max	74.5	71.6	74.5			
Years from diagnosis to screening						
Median	1.1	1.4	1.1			
Min	0.3	0.3	0.3			
Max	7.1	25	25			
Days disease free until randomisation						
Median	216	187	209			
Min	43	94	43			
Max	328	357	357			

	*n*	%	*n*	%	*n*	%
Type of melanoma						
Cutaneous	17	100	16	100	33	100
Cancer Stage at diagnosis						
IIA	0	0.0	1	6.3	1	3.0
IIB	1	5.9	0	0.0	1	3.0
IIC	0	0.0	1	6.3	1	3.0
IIIA	2	11.8	3	18.8	5	15.2
IIIB	4	23.5	4	25.0	8	24.2
IIIC	6	35.3	5	31.3	11	33.3
IV	4	23.5	2	12.5	6	18.2
Prior chemotherapy for advanced melanoma						
Yes	0	0.0	1	6.3	1	3.0
No	17	100	15	93.8	32	97.0
Prior radiotherapy for advanced melanoma						
Yes	8	47.1	5	31.3	13	39.4
No	9	52.9	11	68.8	20	60.6
Prior other cancer						
Yes	2	11.8	1	6.3	3	9.1
No	15	88.2	15	93.8	30	90.9
ECOG						
0	17	100	16	100	33	100

All patients started treatment within two days of the randomisation, and all except one received the planned dose of 10 × 10^6^ DCs for both cycles 1 and 2; one patient in the DCV arm had insufficient yield from the leukapheresis and received 4.3 × 10^6^ DCs at each dose. As release criteria, all products contained > 70% CD83^+^ HLA-DR^+^ cells that were > 70% viable, as determined on a thawed sample prior to injection. Further phenotypic evaluation on thawed samples with a 15 colour antibody panel (Supplementary Table 2) showed that the mature DCV and DCV + *α*-GalCer products were highly homogenous (in contrast to their immature precursors), with the dominant population expressing CD40, CD54, CD80, CD83, CD86, HLA-ABC, HLA-DR, CD1d, CCR7, and PD-L1 (Supplementary Fig. 1).

#### Primary outcome measure: NY-ESO-1 peptide-specific T cell response

**Fig. 2:**
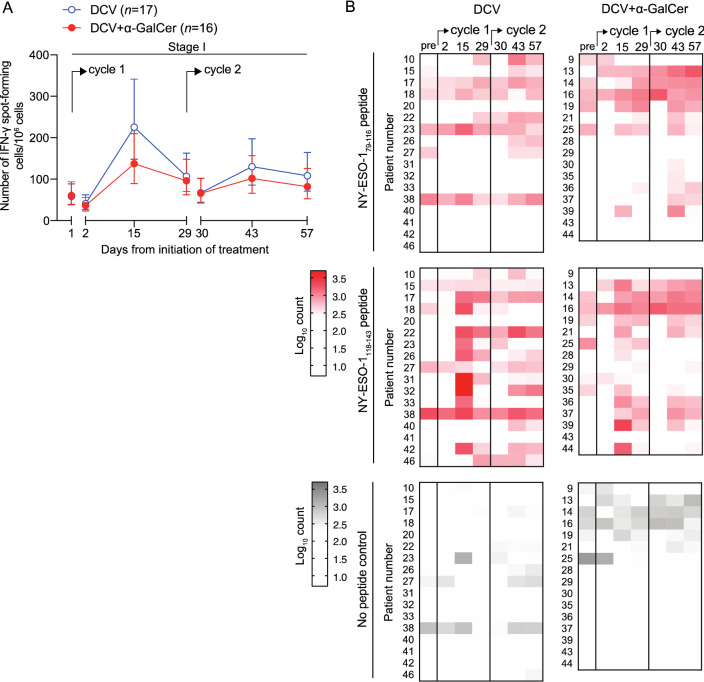
Analysis of NY-ESO-1-specific T cell responses by ex vivo IFN-γ ELISpot at Stage I. **a** Primary analysis showing estimated mean NY-ESO-1-specific IFN-γ spot-forming units (to either peptide) per million PBMCs (± 95% confidence intervals) over time for both treatment arms in Stage I of the study. The difference in AUC for the two treatment arms was not significant (− 6.85, 95% confidence interval − 21.65 to 7.92; *P* = 0.36). **b** Heatmaps of mean log_10_-transformed T cell count for each patient against the different peptides over time (red), with mean background levels in no-peptide control samples shown (grey)

To assess the impact of including *α*-GalCer in the DC-based vaccine format on T cell response, the AUC of mean total count of NY-ESO-1_79–116_ and NY-ESO-1_118–143_-specific T cells in PBMCs before and after vaccination in each of the treatment arms was compared. For each patient a sample collected 1–5 days before treatment and another immediately before each vaccination provided baseline values, while post-vaccination samples were taken 24 h after vaccination, and 2 and 4 weeks after vaccination. Assessment of NY-ESO-1-specific T cell response was by ex vivo IFN-γ ELISpot, with count expressed as number of spot-forming cells per million PBMCs. The geometric mean NY-ESO-1-specific T cell counts at each time point and by treatment arm are shown in Fig. 2a. The difference in mean AUC of T cell count between the treatment arms was not significant (− 6.85, 95% confidence interval − 21.65 to 7.92; *P* = 0.36). The planned sensitivity analysis adjusting for baseline imbalance had negligible impact. The inclusion of *α*-GalCer in the vaccines therefore had no impact on T cell response by this measure.

Heatmaps in Fig. 2b show log_10_-transformed T cell count for each NY-ESO-1 peptide. Changes in T cell count from baseline in response to vaccination were seen to either peptide (and often both) and were observed in the vast majority of patients. Responses to NY-ESO-1_79–116_ tended to peak after the second vaccine, whereas responses to NY-ESO-1_118–143_ tended to peak after the first.

### Secondary efficacy measures

#### T cell response assessed by ICS

The induction of an NY-ESO-1-specific T cell response to vaccination over the first two cycles was classified “yes” or “no” on the basis of detecting IFN-γ-producing cells by ICS following in vitro restimulation of PBMCs for 10 days with the long NY-ESO-1 peptides. Antibodies used and the gating strategy for flow cytometry are provided in Supplementary Table 2 and Supplementary Fig. 2. One patient in the DCV + *α*-GalCer arm was excluded from this analysis due to poor recovery of cells. The proportions of patients with a response were similar between treatment arms, with 86.67% on the DCV + *α*-GalCer arm versus 82.35% on DCV arm (*P* = 1.0, Table 2). Patients who had NY-ESO-1-specific T cell responses were further categorised as to whether the responses were CD4^+^ or CD8^+^ T cell-mediated, or both. The pattern of CD4^+^ and CD8^+^ T cell responses was very similar in the two arms; the majority of patients had only a CD4^+^ T cell response (Table 2).

**Table 2:** Proportions of NY-ESO-specific T cell responders determined by ICS in Stage I

Treatment	Response	*n*	%	95% CI for proportion	*P*-value^b^
DCV (*n* = 17)	All (CD4^+^ and/or CD8^+^)	14	82.35	(56.57 to 96.20)	1.0
DCV + *α*-GalCer (*n*= 15)^a^		13	86.67	(59.54 to 98.34)	
DCV (*n* = 17)	No response	3	17.65	(3.80 to 43.43)	0.7922
	CD4^+^ only	9	52.94	(40.42 to 80.84)	
	CD8^+^ only	0	0.00	NA	
	Both CD4^+^ and CD8^+^	5	29.41	(10.31 to 55.96)	
DCV + *α*-GalCer (*n* = 15)^a^	No response	2	13.33	(1.66 to 40.46)	
	CD4^+^ only	10	66.67	(38.38 to 88.18)	
	CD8^+^ only	0	0.00	NA	
	Both CD4^+^ and CD8^+^	3	20.00	(4.33 to 48.069)	

^a^Patient 2–043 excluded due to insufficient cells

^b^Fisher's exact test (as 50% expected counts < 5)

#### NKT cell response

**Fig. 3:**
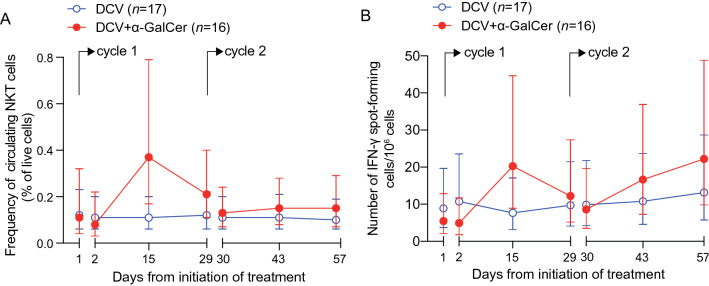
Analysis of NKT cell responses at Stage I. **a** Mean frequency of NKT cells detected by flow cytometry in PBMCs (± 95% confidence intervals) at each time point and by treatment arm. The difference in AUC between treatment arms was not significant (28.74, 95% confidence interval − 5.95 to 63.43; *P* = 0.10) **b** Estimated mean α-GalCer-specific IFN-γ spot-forming cells per million PBMCs (± 95% confidence intervals) detected by ELISpot. The difference in AUC between treatment arms was not significant (17.62, 95% confidence interval -5.97 to 41.21; *P* = 0.1424)

To evaluate the cellular response to α-GalCer, NKT cell counts were measured in PBMCs by flow cytometry using fluorescent α-GalCer-loaded human CD1d tetramers. The gating strategy is provided in Supplementary Fig. 3. Data are expressed as number of NKT cells per 100 CD3^+^ cells. The patient in the DCV + α-GalCer arm with insufficient recovery of cells was also excluded from this analysis. Mean NKT cell count at each time point and by treatment arm is shown in Fig. 3a. The mean AUC was higher in the DCV + α-GalCer arm than in DCV alone, but the difference was not significant (28.74, 95% confidence interval − 5.95 to 63.43; *P* = 0.10). Similar results were found with NKT cells measured by IFN-γ ELISpot using α-GalCer as recall agent (Fig. 3b) (17.62, 95% confidence interval − 5.97 to 41.21; *P* = 0.1424).

**Table 3:** Comparison of cytokine levels for DCV + *α*-GalCer versus DCV arms in Stage I

Cytokine	Difference in AUC	95% confidence interval	*P*-value
IL-4	−0.18	(−5.40 to 5.04)	0.9465
IL-6	−0.58	(−14.98 to 13.83)	0.9370
IL-10	−2.17	(−18.22 to 13.89)	0.7900
IL-12p70	0.74	(−14.87 to 16.34)	0.9257
TNF	−0.87	(−6.93 to 5.19)	0.7779
IFN-γ	2.41	(−4.7 to 9.51)	0.5053
MCP-1	3.75	(−11.43 to 18.93)	0.6266
MIP-1α	−1.13	(−10.55 to 8.29)	0.8127
MIP-1β	1.14	(−7.54 to 9.82)	0.7962
RANTES	−0.34	(−4.95 to 4.27)	0.8832
IP-10	2.38	(−8.33 to 13.09)	0.6613

#### Serum cytokine levels

To evaluate serum cytokine responses to the vaccines in the two treatment arms, levels of 11 cytokines that were specified a priori (based on association with in vivo NKT cell responses in earlier trials) were compared using multiplex immunoassays. Median cytokine levels based on fluorescence intensity are provided in Supplementary Table 3, and changes over time for each patient are presented as heatmaps in Supplementary Fig. 4. The comparisons of the AUCs of the median fluorescence intensity (after subtraction of background fluorescence) are shown in Table 3. There were no statistically significant differences between treatment arms.

### Safety outcomes at Stage I

During leukapheresis procedures to harvest cells for DC generation, which were conducted before randomisation, there were six grade 3 adverse events (AE) reported: infection (1 event); backpain (1); syncope (2); depressed level of consciousness (1) and vasovagal reaction (1). One of the patients, who experienced three of these events, did not complete leukapheresis and withdrew before randomisation. The patient had osteomyelitis, back pain (managed with pain medication) and depressed level of consciousness. Once the SAE sequelae had resolved, the patient re-entered screening and ultimately completed all cycles of treatment without any further AEs. Adverse events experienced in Stage I of the treatment schedule are presented in Supplementary Table 4. No patients experienced a grade 3 or higher AE over the first two cycles of vaccination after randomisation in Stage I of the study.

### Stage II

#### Capacity of repeated DCV + *α*-GalCer to boost NY-ESO-1-specific T cell response

**Fig. 4:**
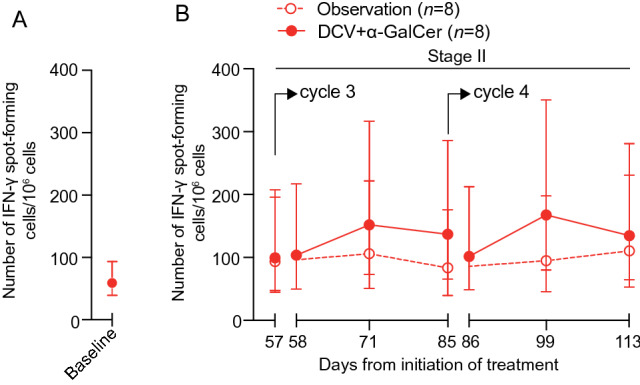
Capacity of repeated DCV + *α*-GalCer to boost T cell response. Estimated mean NY-ESO-1-specific IFN-γ spot-forming cells (± 95% confidence intervals): **a** at baseline for patients in Arm 2 at Stage I, and **b** over time for patients in Arm 2 at Stage II. These patients received two cycles of DCV + *α*-GalCer at Stage I, and at Stage II were randomly allocated to no further vaccination (observation) or two further cycles DCV + *α*-GalCer. The difference in AUC between treatment arms at Stage II was not significant (7.60, 95% confidence interval − 16.18 to 30.31; *P* = 0.55)

To assess whether DCV + *α*-GalCer-induced T cell responses could be boosted by repeated vaccination, the 16 patients on the DCV + *α*-GalCer arm were randomised at Stage II to receive a further two cycles of DCV + *α*-GalCer, or were observed only, thereby enabling analysis of two versus four vaccine cycles. In assessment of mean T cell count by ex vivo IFN-γ ELISpot, the additional impact of vaccine treatment on AUC did not reach statistical significance (Fig. 4) (7.60, 95% confidence interval − 16.18 to 30.31; *P* = 0.55). Responses were also measured by ICS after restimulation on peptide (although one patient in the observation arm had insufficient cells for analysis). The numbers of patients with a response were 8/8 (100%) in the group that received the additional DCV + *α*-GalCer vaccines and 5/7 (71%) in the observation group (*P* = 0.2). There was no evidence of a difference in the AUC for NKT cells measured by flow cytometry between the two arms (2.2, 95% confidence interval − 19.2 to 23.7; *P* = 0.84), or by IFN-γ ELISpot with *α*-GalCer as recall agent (1.4, 95% confidence interval − 30.1 to 32.9; *P* = 0.93). Thus, the benefit to the T cell response of extra cycles of vaccination was marginal, with no evidence of associated NKT cell activity with the additional DCV + *α*-GalCer cycles.

#### Capacity of DCV + *α*-GalCer to boost DCV-induced NY-ESO-1-specific T cell response

**Fig. 5:**
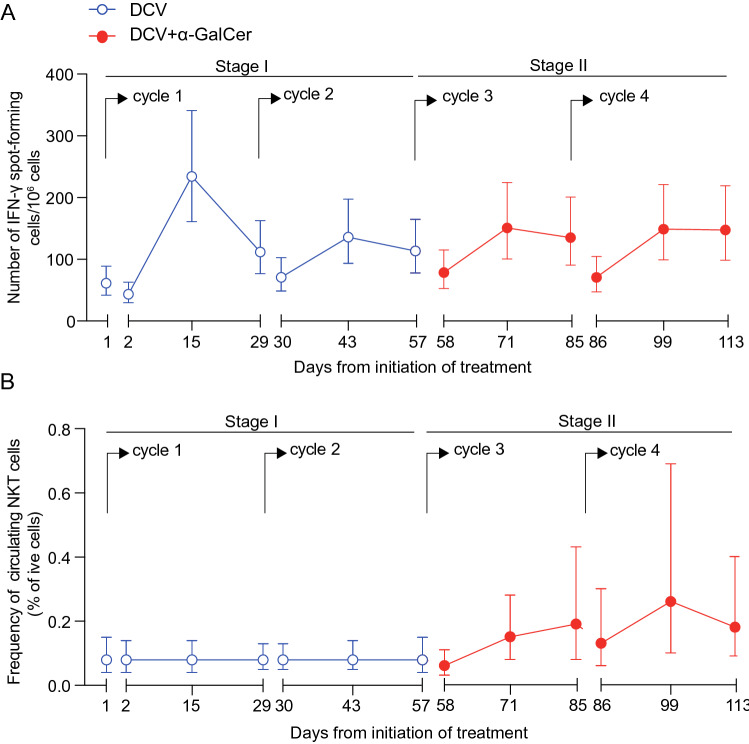
Capacity of DCV + *α*-GalCer to boost DCV-induced NY-ESO-1-specific T cell response. **a** Estimated mean NY-ESO-1-specific IFN-γ spot-forming cells (± 95% confidence intervals) over time for patients in Arm 1 at Stage I and II. These patients had received two cycles of DCV at Stage I, and then crossed over to receive two further cycles DCV + *α*-GalCer at Stage II. The difference in AUC of Stage II over Stage I was not significant (−13.66, 95% confidence interval − 27.62 to 0.30; *P* = 0.06). **b** Estimated mean NKT cell counts per total CD3^+^ cells in the blood, as detected by flow cytometry. All the estimates were translated back to the original scale for ease of interpretation. A Poisson regression was used to compare AUC at the different stages. The difference in AUC of Stage II over Stage I was statistically significant (40.09, 95% confidence interval: 8.9 to 71.28;* P* = 0.012)

To assess whether vaccines with *α*-GalCer could contribute to a significant boost to responses stimulated initially by vaccines without *α*-GalCer, 15 patients on DCV alone for the two cycles in Stage I crossed over to receive two cycles of DCV + *α*-GalCer at Stage II. No significant difference in AUC for T cell count assessed by IFN-γ-ELISpot was observed between the two treatment stages for these patients (Fig. 5a) (− 13.66, 95% confidence interval − 27.62 to − 0.30; *P* = 0.06). In contrast, for NKT cells counts measured by flow cytometry, the AUC was significantly higher after the cross-over, indicating NKT cells proliferated in response to DCV + *α*-GalCer (40.09, 95% confidence interval: 8.9 to 71.28; *P* = 0.012). The cytokine responses were also evaluated in the cross-over. Only RANTES was significantly increased after cross-over (Supplementary Table 5), although this result should be interpreted with caution due to small sample size, and the levels of this cytokine were not normally distributed. Thus, while there was some evidence of NKT cell activity with the DC + *α*-GalCer vaccines used in the boost, this did not contribute to a stronger NY-ESO-1-specific T cell response than had been initiated with DCV.

#### Safety outcomes at Stage II

Adverse events experienced in Stage II of the treatment schedule are presented in Supplementary Table 6. No patients experienced a grade 3 or higher adverse event over the 3rd and 4th cycles of vaccination.

### Clinical course and subsequent therapy

**Fig. 6:**
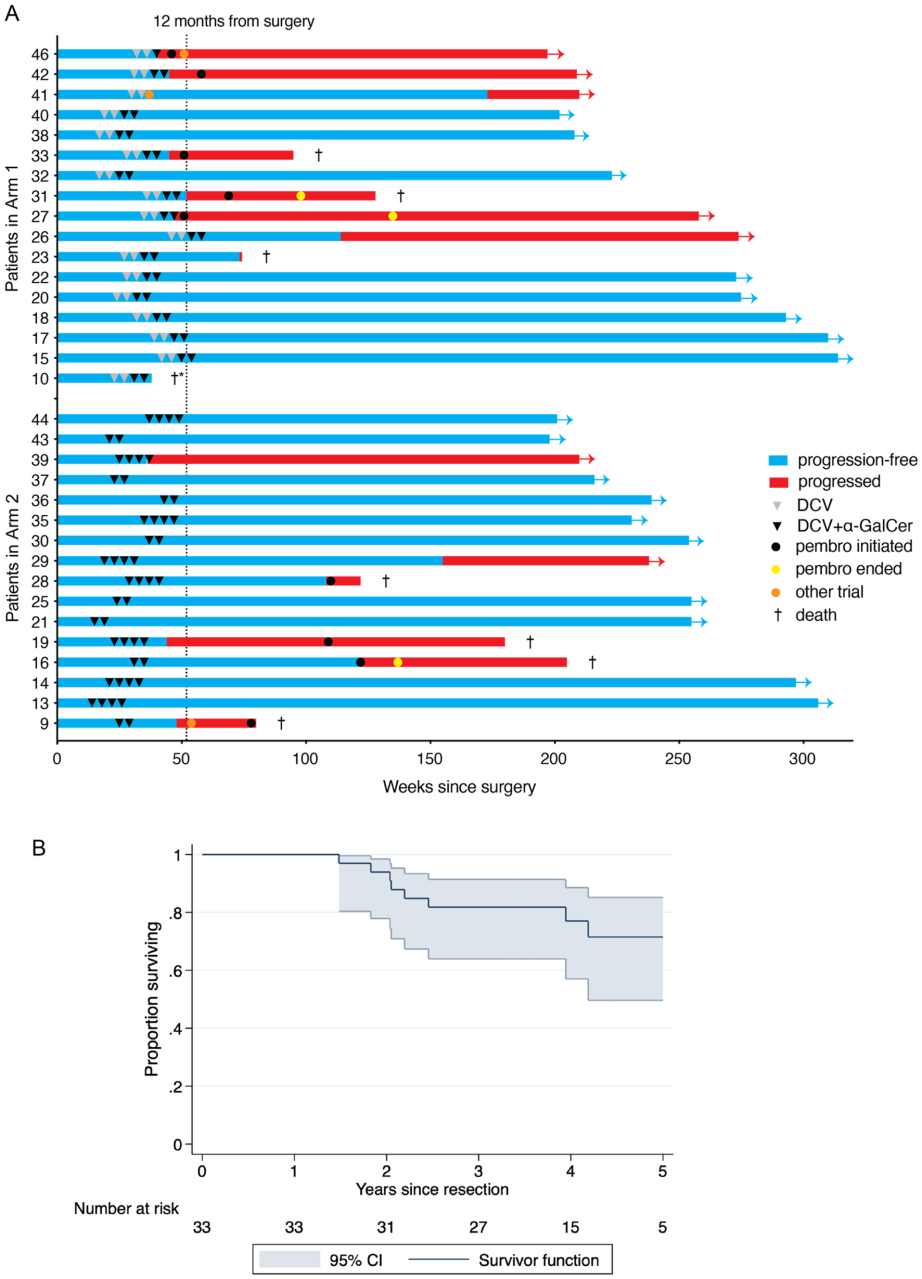
Patient treatment, clinical course and survival. **a** Swimmer plot of clinical course for each patient. All patients that were randomised at Stage I initiated therapy within 12 months of surgical resection, indicated by dotted line. Times when patients progressed or were subject to further treatment are indicated, notably checkpoint blockade with pembrolizumab (pembro). *Patient 10 died of cause unrelated to disease progression. **b** Patient survival from their most recent resection, with 95% confidence intervals indicated

A swimmer plot in Fig. 6a depicts the clinical course of individual patients in the study. All patients received at least two cycles of vaccine, and all but one patient received at least one vaccine with *α*-GalCer. Of the 33 high-risk patients enrolled, 14 progressed, of whom 7 have died. Another died from an unrelated cause. The remainder have controlled disease, in some cases with further treatment involving immune checkpoint blockade with pembrolizumab (3 patients) or systemic therapy on another trial (1 patient). The 3-year survival rate from their most recent resection was 81% (95% confidence interval 63.9–91.4%) (Fig. 6b), but we note this will be biased up relative to a clinical cohort post-resection as the study participants had to have survived long enough to be recruited into the study (the median time from resection to first vaccination was 6.9 months).

## Discussion

In this randomised study, fully resected high-risk melanoma patients were assigned to receive autologous DC-based vaccines loaded with long peptides from NY-ESO-1 alone, or the peptides loaded together with the NKT cell agonist *α*-GalCer. While T cell-mediated responses to the peptides in the vaccine were observed in the vast majority of patients, the inclusion of *α*-GalCer to recruit NKT cell help, which we anticipated would enhance the response, did not have a significant impact on T cell levels induced.

The vaccine-induced T cell responses observed could be detected in PBMCs evaluated directly ex vivo by IFN-γ ELISpot, using the long NY-ESO-1-derived peptides as recall agents. There are only a few other reports where NY-ESO-1-specific T cell responses have been detected so readily without requiring several days of in vitro restimulation with antigen [26, 27]. Analyses were also conducted on data collected by ICS following 10 days of in vitro restimulation on peptide, which enabled us to determine whether responses were CD4^+^ or CD8^+^ T cell-mediated. Responses of either type, or both, were detected by this technique in 86.67% of patients that received vaccines with *α*-GalCer (13 of 15 patients evaluated), and 82.35% that received vaccines without *α*-GalCer (14/17). Overall, CD4^+^ T cell-mediated responses predominated (27/32 patients evaluated) with reactivity to either peptide, with no significant impact on this trend associated with the inclusion of *α*-GalCer.

The lack of significant effect of including *α*-GalCer in the vaccine on the circulating T cell response is at odds with numerous preclinical studies conducted with DCs in vivo in mice, where significant improvements in T cell response have been observed [16, 28–30]. In generating vaccines for clinical use, we considered carefully whether to inject immature or mature DCs. While immature DCs were more likely to be effective recipients of NKT cell-mediated help, injection of immature cells into volunteers has previously been shown to result in the induction of regulatory T cells [31], which could be clinically detrimental. We therefore elected to use cytokine-mature DCs, a decision supported by our preclinical studies in mice, where bone marrow-derived DCs loaded with antigen and *α*-GalCer could enhance antitumour responses irrespective of maturation status. The added rationale was that even if NKT cell help failed, the mature product would give the best chance of eliciting an NY-ESO-1-specific T cell response that could potentially benefit the patient [32]. In fact, the mature product, even without *α*-GalCer, was an effective vaccine platform for generating NY-ESO-1-specific T cell responses in the majority of patients. In secondary analysis, the T cell responses induced with DCV could be boosted within the same patients with DCV + *α*-GalCer, but the responses were not superior in size, as might have been expected if *α*-GalCer was functioning as a powerful immune adjuvant. In further secondary analyses, it was shown that multiple rounds of DCV + *α*-GalCer (comparing four versus two cycles) did not significantly improve T cell responses. However, it should be noted that our analyses were limited to the blood, and it remains possible that including *α*-GalCer may improve functionality and distribution to other tissues. For example, our own preclinical studies with synthetic *α*-GalCer-peptide conjugates designed to target APCs in vivo showed that NKT cell activation can facilitate accumulation of resident memory CD8^+^ T cells in the liver [33].

A likely explanation for the lack of T cell enhancement with *α*-GalCer in the vaccine is that the NKT cell responses induced in the patients were insufficient to provoke the helper responses required. This could either be a consequence of low NKT cell frequency, poorly presented agonist, or lack of functional capacity. There was more variability in NKT cell count in the DCV + *α*-GalCer arm relative to the DCV arm (where NKT cells should be unaffected), with individual cases where increases in NKT cell populations to DCV + *α*-GalCer were observed. Nonetheless, the AUC for NKT cell count of DCV + *α*-GalCer arm did not reach statistical significance over levels in the DCV group. The only analysis of NKT cell activity that reached statistical significance was an increase in NKT cell frequency in patients who crossed over from receiving DCV to DCV + *α*-GalCer. This was not associated with an increase in mean NY-ESO-1-specific T cell response, although one patient who had failed to induce a peptide-specific response to DCV did induce a response after cross-over to DCV + *α*-GalCer. However, this induced response could reflect the effect of repeated antigen dosing rather than result from the addition of *α*-GalCer.

It is possible that NKT cell function was impaired in the patient cohort. There have been previous reports of NKT cell dysfunction in cancer patients [34–38], including in melanoma [39]. Prior treatments have also been reported to have a negative impact on NKT cells, including radiation treatment [39], which some patients in our study had received. However, the patients were disease-free at the time of treatment, and there had been a minimum “wash-out” period of 6 weeks from radiation treatment before vaccination. One of the prime readouts of NKT dysfunction has simply been reduced numbers compared to healthy controls. However, the range of NKT cell frequencies observed at baseline in our study was not remarkably different to healthy donors of similar age in a previous study from our group [40].

It should be noted that the modest levels of NKT cell proliferation observed in this trial are in contrast to a comparable study conducted by Chang et al., with *α*-GalCer-pulsed MoDCs (without peptides), prepared and matured in a similar manner [19]. They observed > 100-fold expansion of the peripheral NKT cell population in all 5 patients studied and could detect these up to 6 months after vaccination. Furthermore, the NKT cell activation correlated with an increase in serum levels of IL-12p40, IP-10 and MIP-1β after 24 h of injection. These changes were not observed when the patients in the study were first injected with DCs without *α*-GalCer. In our study, increases in IP-10 were observed in response to vaccination, and while we did not specifically look at IL-12p40, we did see increases in IL-12p70. However, these increases were not unique to the DCV + *α*-GalCer vaccinated group, with similar cytokine levels seen in patients in the DCV arm. Our interpretation of our phase I study, where only DCV + *α*-GalCer were used, needs to be reconsidered in this light; the cytokine responses observed may only have been a consequence of the mature MoDCs used, and not reflect NKT cell activation.

Given that effective NKT cell helper function in animals is typically associated with extensive NKT cell proliferation, the weak proliferation observed in vivo here was perhaps insufficient to meet the threshold required for adjuvant activity. The dose of *α*-GalCer loaded onto the MoDCs may have been a factor, although this was selected based on literature precedent where proliferation had been observed clinically [19]. In preparing the GMP grade material, the compound was dissolved in DMSO, filtered for sterility and stored frozen. Assessment by analytical HPLC showed there was no loss of material in the filtration process and re-thawed samples were not subject to any observable decomposition or loss of potency. The thawed glycolipid was capable of stimulating NKT cells in vitro, as were vaccines prepared over the course of the study. However, we cannot rule out the possibility that the bioavailability of *α*-GalCer was ultimately suboptimal in our study, noting that a formulation matrix for dissolving the glycolipid has been used in previous clinical studies [41].

The NY-ESO-1 antigen used in this study has been targeted by vaccines in several previous clinical studies. In a retrospective analysis of early phase clinical trials of adjuvant NY-ESO-1-based vaccine immunotherapy (using DCs, peptide in adjuvant, or peptides with toll-like receptor agonists), Lattanzi et al. compared 67 vaccinated patients with resected stage III melanoma to 123 historical control patients who received no adjuvant therapy and found that the vaccine was associated with decreased risk of recurrence and death [42]. In contrast, Cebon et al. assessed the impact of vaccination with NY-ESO-1 protein in ISCOMATRIX in 56 resected stage IIc, IIIb, IIIc and IV melanoma patients compared to 51 patients that received ISCOMATRIX alone and found no impact on survival endpoints despite evidence of induced adaptive responses [43]. The authors suggested that immune escape through the downregulation of NY-ESO-1 and/or HLA class I molecules on the tumour may have contributed to relapse. Although our study was not designed to evaluate clinical efficacy, it remains possible that the induction of NY-ESO-1-specific T cells combined with an NKT cell response (albeit weak) could have provided some clinical benefit. In this context, it is important to note that animal studies have shown that *α*-GalCer-stimulated NKT cells can be useful antitumour effectors in addition to conventional T cells. In fact, it was structure activity studies of glycolipids with antitumour activity from the Okinawan marine sponge *Agelas mauritianus* that resulted in the design of *α*-GalCer as a synthetic chemical entity with potent activity in animals [44–46]. To date, 25 of the 33 high-risk melanoma patients in this trial have survived, as have 7 of the 8 patients from our previous phase I trial, with some controlling disease relapse with further treatment, notably checkpoint blockade. All but one of these patients received NY-ESO-1 peptide-loaded vaccines with *α*-GalCer at some point in their treatment. It remains unclear whether this unexpected but encouraging clinical outcome reflects the impact of a valuable immune response, or a selection bias. In this context, the median timeframe from surgical resection to first vaccination was 6.9 months, so individuals with a rapid disease relapse were likely underrepresented in the enrolled cohort.

Overall, this study failed to demonstrate a significant advantage to loading peptide-pulsed MoDCs with* α*-GalCer to enhance circulating peptide-specific T cell responses. Although this may reflect inability to recruit the helper function of NKT cells, especially as only limited NKT cell activity was observed in this study cohort, it remains possible that there were changes in T cell distribution to other tissues that were not investigated. The study did show that it is possible to achieve a high population coverage of NY-ESO-1-specific responses with the strategy of incorporating long peptides covering multiple epitopes into the vaccine. Additionally, all of the autologous products were prepared without issue and injected with a good safety profile.

### Supplementary Information

Below is the link to the electronic supplementary material.

Supplementary file1 (PDF 3362 KB)

## Abbreviations

*α*-GalCer: *α*-Galactosylceramide
