# Supplementary material for: A randomised controlled trial of long NY-ESO-1 peptide-pulsed autologous dendritic cells with or without alpha-galactosylceramide in high-risk melanoma
Source: Cancer Immunol Immunother. 2023 Mar 7;72(7):2267–82. doi: 10.1007/s00262-023-03400-y (PMC10264280; doi:10.1007/s00262-023-03400-y)
Supplement: Supplementary file 1 — Supplementary file1 (PDF 3362 KB) [file 262_2023_3400_MOESM1_ESM.pdf]

SUPPLEMENTARY MATERIAL

A randomized controlled trial of NY-ESO-1 peptide-pulsed autologous dendritic cells with or without alpha-galactosylceramide in high-risk melanoma

Nathaniel Dasyam, Katrina J. Sharples, Catherine Barrow, Ying Huang, Evelyn Bauer, Brigitta Mester, Catherine E. Wood, Astrid Authier-Hall, Marina Dzhelali, Tess Ostapowicz, Rajiv Kumar, Jessica Lowe, Alice Maxwell, Olivia K. Burn, Geoffrey M. Williams, Sarah E. Carley, Graham Caygill, Jeremy Jones, Susanna T.S. Chan, Victoria A. Hinder, Jerome Macapagal, Monica McCusker, Robert Weinkove, Margaret A. Brimble, Gavin F. Painter, Michael P. Findlay, P. Rod Dunbar, Olivier Gasser, Ian F. Hermans.

Includes:

Supplementary Materials and Methods

Supplementary Fig. 1	Gating strategy for assessing intracellular cytokine staining on T cells.
Supplementary Fig. 2	Gating strategy for NKT cells.
Supplementary Fig. 3	High dimensional flow cytometry analysis of immature DC and mature vaccine products.
Supplementary Fig. 4	Heatmaps of cytokine induction for each patient.
Supplementary Table 1	Antibody panels used for flow cytometry
Supplementary Table 2	Baseline haematology, biochemistry and coagulation by treatment group (Stage I).
Supplementary Table 3	Cytokine levels in serum (pg/ml) in Stage I.
Supplementary Table 4	Adverse events post randomisation to end of Stage I.
Supplementary Table 5	Comparison of cytokine levels for Arm 1 cross-over from DCV for cycles 1 and 2 at Stage I to DCV+α-GalCer for cycles 3 and 4 at Stage II.
Supplementary Table 6	Adverse events at Stage II. .

Supplementary Methods

Antigenic peptides and α-GalCer

The DCs were loaded with two long peptides from NY-ESO-1 (NY-ESO-1_{79-116}, GARGPESRLLEFYLAMPFATPMEAELARRSLAQDAPPL and NY-ESO-$1_{118-143}$, VPGVLLKEFTVSGNILTIRLTAADHR). As an approach to increase likelihood of measuring T cell responses to evaluate the impact of α-GalCer, HLA class I-binding peptides from influenza proteins were loaded separately onto half of the cells of the vaccine with or without α-GalCer; these peptides were from influenza polymerase basic protein 1 (PB-$1_{489-497}$, TFEFTSFFY), influenza virus matrix 1($M1_{58-66}$, GILGFVFTL), and influenza virus nucleoprotein ($NP_{265-273}$, ILRGSVAHK). However, as none of the participants in the earlier phase I study had detectable MHC class I-restricted $CD8^+$ T cell memory responses to these peptides, and the vaccine did not prime detectable *de novo* $CD8^+$ T cell responses [12], it was anticipated that this readout would be uninformative in the randomised study, and evaluation of responses to these peptides was not included in the statistical plan for the formal endpoints of this study. Good manufacturing practice (GMP) processes were used to generate the peptides (University of Auckland, Auckland, New Zealand) and α-GalCer (GlycoSyn, Lower Hutt, New Zealand).

Vaccine production

The generation of MoDCs and antigen pulsing was conducted under GMP conditions (Malaghan Institute of Medical Research). A Lymphoprep density gradient (Axis Shield, Oslo, Norway) was used to enrich PBMCs from leukapheresis product, with a 20 % sucrose gradient (Calbiochem, Billerica, MA) then used to remove platelets. Monocytes were enriched by adherence to plastic in complete medium consisting of RPMI 1640 medium (Gibco, Life Technologies, Carlsbad, CA) supplemented with 2 % autologous plasma. The adherent fraction was incubated in complete medium overnight, and then maintained in complete medium supplemented with 1,000 U/ml recombinant human granulocyte maturation colony stimulating factor (rhGM-CSF; Genzyme, Lynnwood, Australia) and 1,000 U/ml rh interleukin (IL)-4 (Gibco CTS, Life Technologies) to promote differentiation to monocyte-derived DCs, with cytokines replenished on day 3. On day 5, samples of immature DCs were collected and stored at -20 °C for later *in vitro* studies. The remaining DCs were pulsed with peptides and α-GalCer in complete medium supplemented with a cytokine cocktail of 1,000 U/mL IL-1β (CellGenix, Freiberg, Germany), 1,000 U/mL IL-6

(Gibco CTS), 1,000 U/mL TNF (Gibco CTS) and 1 μg/mL PGE_2 (Cayman Pharma, Neratovice, Czech Republic). To avoid immunodominance of influenza-specific responses over those for the tumour antigen, one half of the cells were incubated with 10 μM of each of the NY-ESO-1 peptides and the other half with 10 μM of each of the influenza peptides. The peptide-supplemented cells were split again at a ratio of 2:1, with the larger fraction supplemented with 100 ng/mL α-GalCer (thereby providing the larger number of α-GalCer-pulsed DCs required for the dosing regimen). On day 6, the separate cultures were washed to remove excess antigens, with the influenza and NY-ESO-1 peptide-pulsed cells then combined at a ratio of 1:1 to give the DCV, and the influenza and NY-ESO-1 peptide-pulsed cells with α-GalCer combined at a ratio of 1:1 to give DCV+α-GalCer. Products were released if the final overnight culture medium was negative for bacterial growth in BacT/Alert FN Plus and BacT/Alert FA Plus cultures (bioMérieux, Marcy-l'Étoile, France) and endotoxin levels were <0.5 EU/mL (Kinetic-chromogenic *Limulus* amebocyte lysate test; Charles River, Melbourne, Australia). The vaccines were cryopreserved in 90 % autologous plasma and 10 % DMSO (OriGen Biomedical, Austin, TX) in 2 mL CellSeal closed-system cryogenic vials (Cook General BioTechnology, Indianapolis, IN) using a controlled rate freezer (Thermo Fisher Scientific). Released products contained >70 % $CD83^+$ HLA-DR^+ cells and were >70 % viable as determined by flow cytometry on a thawed sample.

Vaccine administration

Cryopreserved vaccines were transported in a dry shipper containing liquid N_2 and thawed immediately prior to injection using a drybath at 37 °C at the bedside. Intradermal test doses consisting of 1 x 10^5 cells of the assigned vaccine were administered to ensure no evidence of an immediate antigen-related wheal and flare reaction over 15 min, with injection of the autologous cryopreservation medium alone as control. The vaccine dose consisting of 10 x 10^6 cells was then administered intravenously via a cannula over 1 min, with the patients kept in the ward for 6 h to be monitored.

Analysis of immune response

IFN-γ ELISpot assay for peptide-specific T cells and NKT cells

For the detection of interferon (IFN)-γ-producing T cells and NKT cells in blood, ELISpot plates (Millipore) were pre-coated in-house with 1 μg/mL of anti-IFN-γ antibody (mAb 1-DK1, Mabtech, Nacka Strand, Sweden) in 100 μL of PBS per well at 4 °C overnight. The

next day the plates were washed four times with 200 µL sterile PBS per well. Cryopreserved PBMCs were thawed and washed three times in RPMI 1640 without supplements and then 1-2 x 10^5 live cells were resuspended in fresh AIM-V medium (Gibco) and cultured overnight at 37 °C, 5 % CO_2 in the presence of either 10 µM of the individual peptides and 0.5 ng/mL rhIL-7 to quantify antigen-specific T cells by IFN-γ ELISpot [47], or with 100 ng/mL α-GalCer to quantify NKT cells by IFN-γ ELISpot, in each case to a final volume of 150 µL per well. Analyses were conducted in triplicate, with five medium-only negative controls. As positive controls, additional samples at each timepoint were stimulated with 5 µg/mL phytohemagglutinin (PHA; ThermoFisher Scientific). After incubation, the plates were washed four times with 200 µL PBS per well, and then 100 µL of 1 µg/mL of anti-human IFN-γ biotinylated antibody (mAb 7-B6-1) and plates were incubated for an hour at 37 °C, 5 % CO_2. Plates were then washed again four times and 100 µL of 1:1000 diluted streptavidin-ALP was added to each well and incubated at room temperature for 45 min away from light. Plates were then washed six times with PBS, and 100 µL of BCIP/NBT-plus substrate (Mabtech) added to each well for spot development. The reaction was stopped by washing wells six times, using 250 µL H_2O per well. Numbers of IFN-γ-producing spots determined using an AID reader (Autoimmun Diagnostika GmbH, Strassberg, Germany). For data visualisation of ELISpot data using heatmaps, mean $\log_{10}$-transformed T cell count from technical replicates were plotted to each peptide over time for all treated patients. To avoid zero values, $\log_{10}(x+a)$ was used instead of $\log_{10}(x)$, where "a" was 0.7 - the minimum required after considering the entire data set.

Intracellular cytokine staining (ICS) and detection

Thawed PBMCs from each timepoint were washed as above and incubated in triplicate with 10 µM of each peptide for 10 days in RPMI 1640 supplemented with 5 % AB serum (Sigma-Aldrich, St. Louis, MO) and 50 U/mL IL-2. The cells were then restimulated overnight with the same peptide in the presence of anti-CD28/CD49d (BD Biosciences), 0.3 µg/mL monensin and 0.5 µg/mL brefeldin A (both Sigma-Aldrich). The cells were first washed twice with 200 µL PBS, then incubated with Live/Dead Fixable Blue staining reagent (ThermoFisher, Waltham, MA) for 10 min. Cell were washed with 200 µL of flow buffer, comprising PBS supplemented with 5 % fetal calf serum and 0.6 mg/mL human normal immunoglobulin (Intragam P, CSL Behring Pty Ltd, Australia) and then stained in 50 µL of an antibody mixture containing antibodies for CD3, CD4, CD8, CCR7, and CD45 RA diluted

in flow buffer. Staining was performed for 10 min at room temperature in the dark. The cells were fixed and permeabilized using Cytofix/Cytoperm (BD Biosciences) and stained with antibodies for IFN-γ, TNF and IL-2 for 20 min in the dark. Antibody fluorophore, identifier, source and dilution are given in **Supplementary Table 2**. After staining, cells were resuspended in a 1:1 mix of 4 % formalin and PBS and kept on ice in the dark before analysis, which was performed on a BD LSR II flow cytometer (BD Biosciences, San Jose, CA). Machine set up including PMT voltages were performed by the core facility (Hugh Green Cytometry Centre) with data analyzed using FlowJo v9.9.6 software using the gating strategy in **Supplementary Fig. 2**.

Analysis of NKT cells by flow cytometry

Samples of PBMCs from each timepoint were assessed in triplicate to determine the number of NKT cells within the T cell fraction by flow cytometry using fluorescent α-GalCer-loaded human CD1d tetramers (ProImmune, Oxford, UK) and antibody to CD3 (clone UCHT1; BD Biosciences, San Jose, CA). Antibody fluorophore, identifier, source and dilution are given in **Supplementary Table 2.** The cells were rested in RPMI medium supplemented with 2 % human AB serum (Sigma) and 1 % Penicillin/Streptomycin (Gibco) before staining was conducted in PBS (Gibco) supplemented with 2 % fetal calf serum and 0.6 mg/mL human normal immunoglobulin (Intragam P, CSL Behring Pty Ltd, Australia). Live/Dead fixable blue stain was used as a viability dye as above. Plates were then washed with flow buffer, and stained with the appropriate antibodies as indicted in **Supplementary Table 2** in a final volume of 50 μL. The cells were then washed twice with flow buffer and fixed in 4 % formalin:PBS before analysis was performed on a BD LSR II flow cytometer with data analyzed using FlowJo v9.9.6 software and the gating strategy in **Supplementary Fig. 3**.

Analysis of cytokines

Serum was tested for increases in the levels of 11 cytokines specified *a priori* based on earlier studies [19–22]: IL-4, IL-6, IL-10, TNF, IL-12p70, IFN-γ, MCP-1, MIP-1α, MIP-1β, and IP-10 were analyzed in triplicate by multiplex immunoassays (Bio-Plex Pro Human Cytokine 27-plex) according to the manufacturer's instructions (BioRad, Hercules, CA), and analysis of RANTES was by ELISA (R&D systems, Minneapolis, MN). The assay output was the median fluorescence intensity (MFI) per bead for each bead classifier. All the negative MFI values, where a cytokine's florescence level was lower than the background,

were set to zero. Heatmaps were generated using Z-scores, data was separated by patient number and then further stratified based on cytokine before Z-scores were calculated. This was done using the formula Z-score = $(x - \mu) / \sigma$; where x = an individual MFI replicate value, μ = the mean and σ = the standard deviation, of all the replicates across all the time points for each cytokine. This was performed in an iterative manner for each patient's cytokine data using R software, and finally visualized using Prism software.

Supplementary Figures and Tables

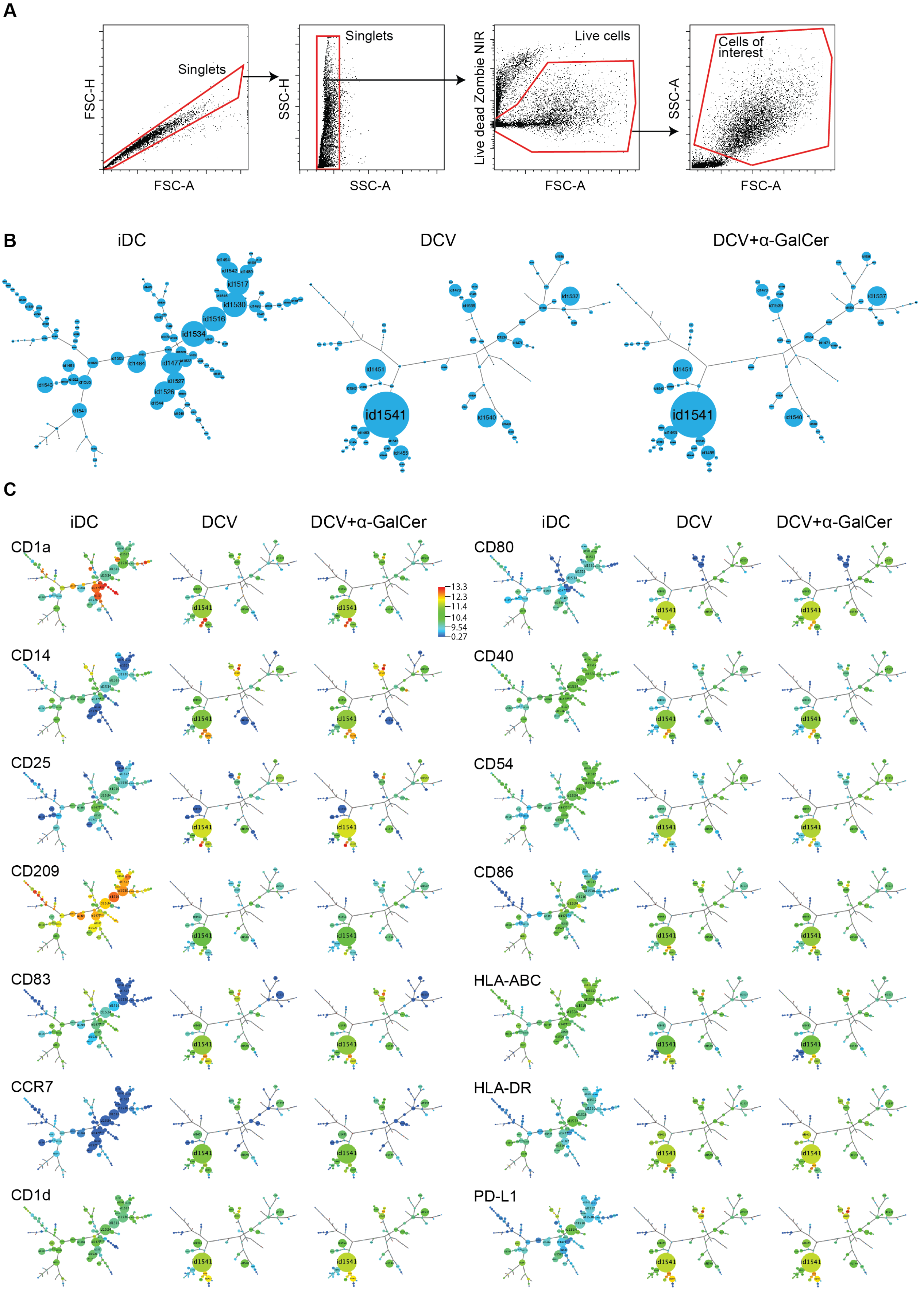

Supplementary Fig. 1 High dimensional flow cytometry analysis of immature DC and mature vaccine products. Samples of immature DCs and excess vaccine (DCV and DCV+α-GalCer) from each patient were thawed and analysed by flow cytometry for expression of 14 cell surface markers. (**A**) Gating strategy. Antibody fluorophore, identifier, source and dilution are given in **Supplementary Table 2**. Cells were stained with Zombie NIR to allow dead cell exclusion before blocking Fc receptors with 2 mg/mL polyclonal human IgG (Intragam P, NZBlood, CSL, Melbourne) for 10 min prior to surface staining. Cells were then stained with surface antibodies for 15 min in buffer containing 2 mg/ml human IgG and Brilliant Buffer Plus (BD Biosciences, at a 1:5 ratio), washed with buffer (PBS + 5 % FBS) and fixed in 1:1 4 % formalin:PBS before analysis. All flow cytometry was performed on a Cytek Aurora Spectral Analyser (Cyte Biosciences). Appropriate fluorophore-labeled antibodies bound to anti-mouse Ig-coated particles (BD Compbeads; BD Biosciences) or single-stained cells were used to perform instrument set up and compensation. Data analysis was performed using FlowJo v9.9.6 software (Tree Star, Inc.) excluding dead cells and doublets by gating. (**B**) Data from iDC, DVC and DCV+α-GalCer were concatenated for generating minimum spanning trees to define clusters of phenotypic similarity using the x-shift algorithm, park of the vortex package in R [48].Thirty iterations with K value increments of 5 were performed, going from 150 to 5, followed by elbow point validation to identify the ideal K value (cluster number), which was identified as K30 with 107 unique clusters. (**C**) Expression of individual markers on the clusters. Dominant cluster id1541 in the mature products expresses high levels of CD40, CD54, CD80, CD83, CD86, HLA-ABC, HLA-DR, CD1d, CCR7, and PD-L1

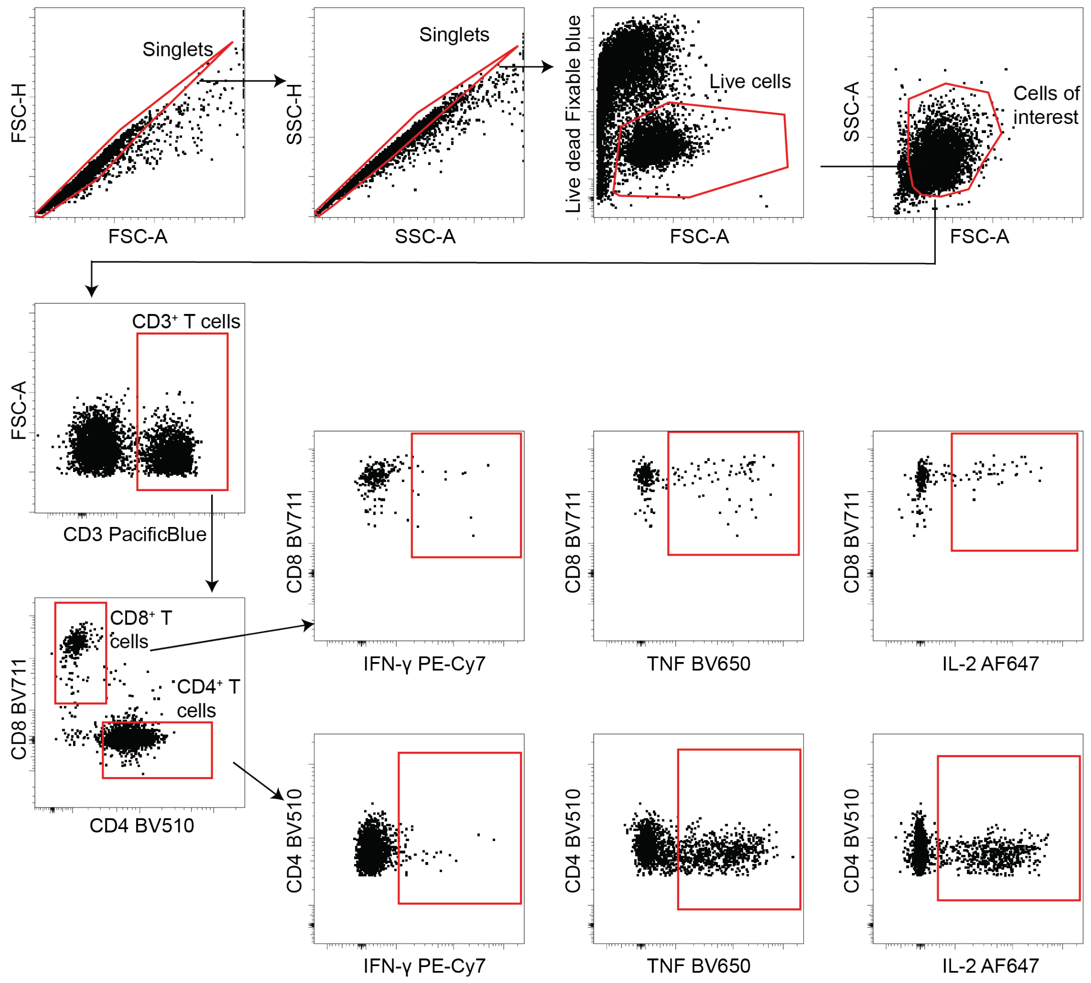

Supplementary Fig. 2 Gating strategy for assessing intracellular cytokine staining on T cells. The gating example shown is on patient-derived PBMCs cultured for 10 days and restimulated with PHA overnight. After gating on singlets, live cells and cells of interest (the gate was set in the lymphocytes region using the scatter profile of the cells), the strategy shown was used to gate $CD8^+$ T cells ($CD3^+$ $CD8^+$ cells) and $CD4^+$ T cells ($CD3^+$ $CD4^+$ cells). The frequency of each T cell subset positive for intracellular IFN-γ, TNF-α and IL-2 was then assessed

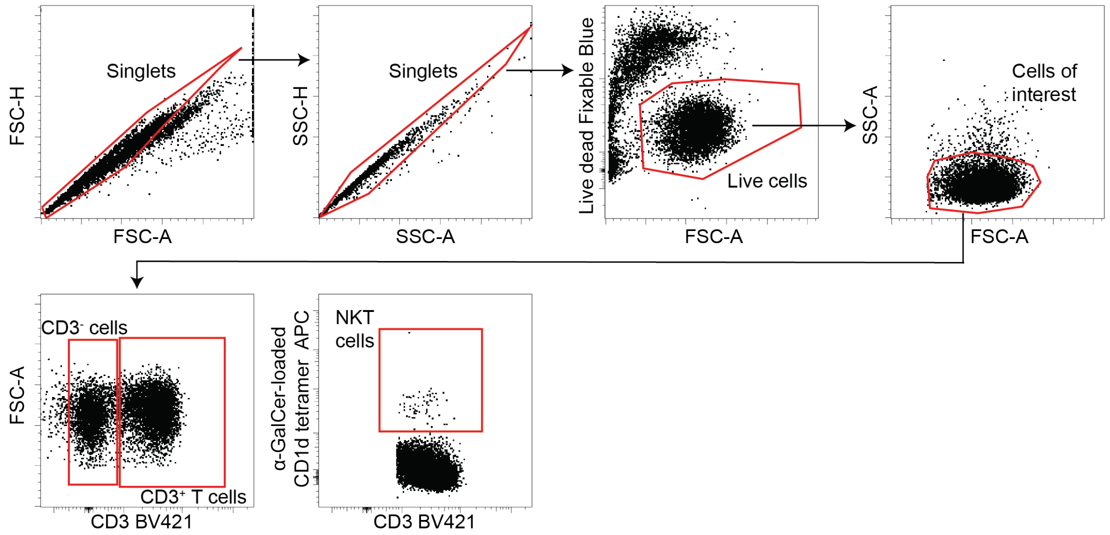

Supplementary Fig. 3 Gating strategy for NKT cells. The gating example shown is on thawed patient-derived PBMCs. After gating on singlets, live cells and cells of interest (the gate was set in the lymphocytes region using the scatter profile of the cells), the strategy shown was used to gate NKT cells (CD3^{+} CD1d tetramer^{+} cells)

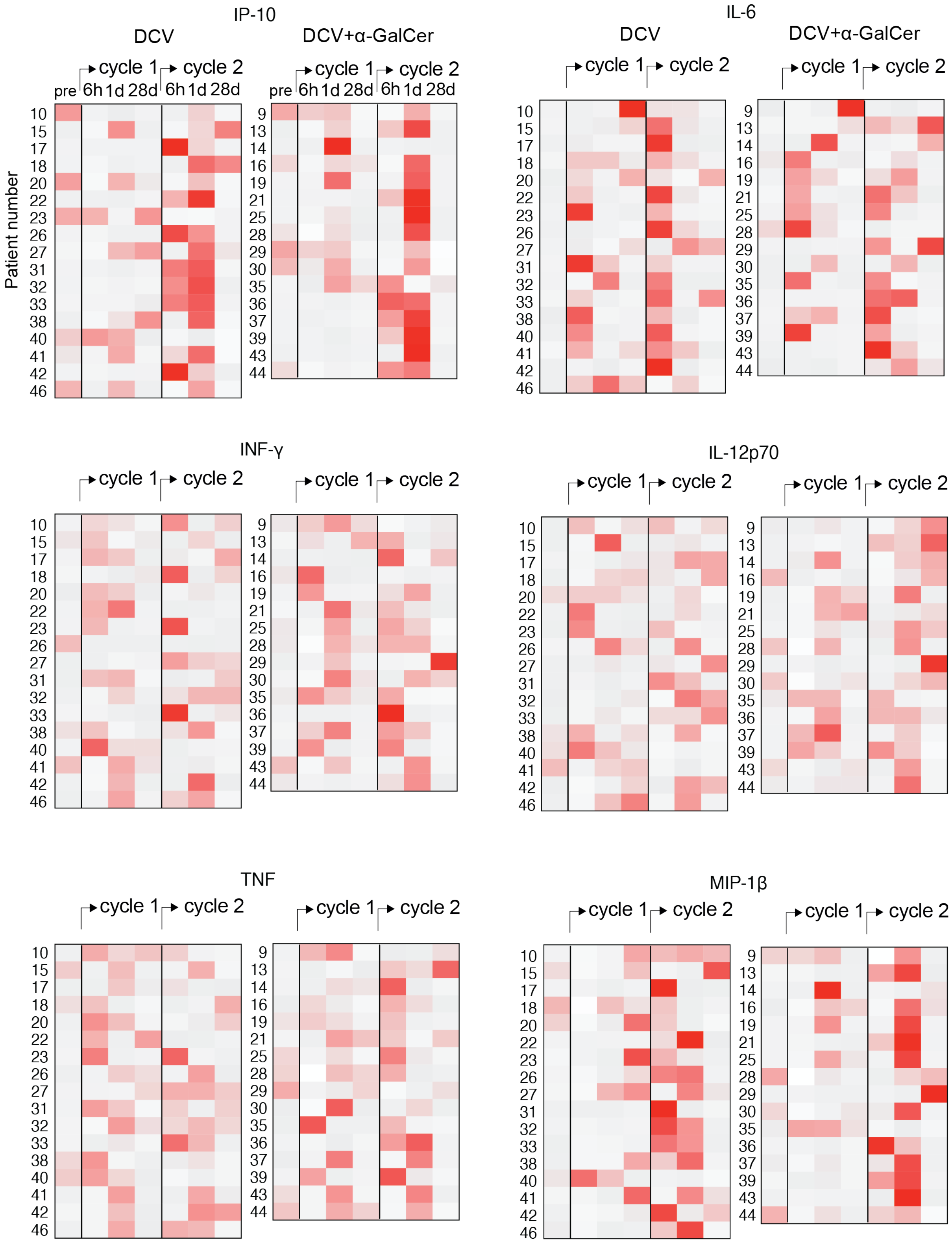
IP-10
DCV
DCV+α-GalCer
cycle 1
cycle 2
pre
6h 1d 28d 6h 1d 28d
Patient number
10 15 17 18 20 22 23 26 27 31 32 33 38 40 41 42 46
9 13 14 16 19 21 25 28 29 30 35 36 37 39 43 44
IL-6
INF-γ
IL-12p70
TNF
MIP-1β

cont'd

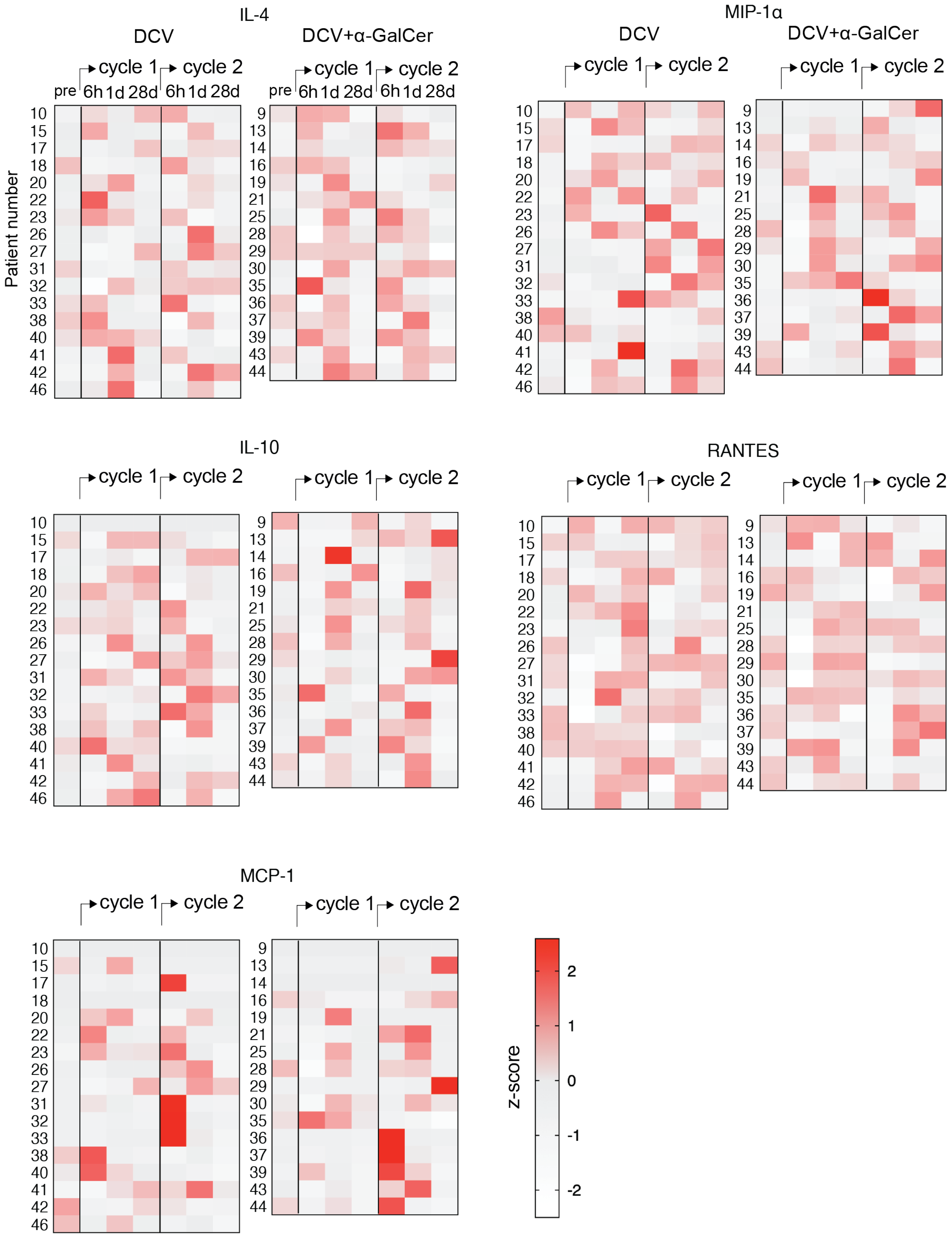

Supplementary Fig. 4 Heatmaps of cytokine induction for each patient. Heatmaps showing change in cytokine with for each patient, conducted as described in Supplementary Methods

Supplementary Table 1 Baseline haematology, biochemistry and coagulation by treatment group (Stage I)

	DCV (n=17)					DCV+α-GalCer (n=16)				
	Median	IQR	Range	No. below normal	No. above normal	Median	IQR	Range	No. below normal	No. above normal
**Haematology**										
Hb (g/L)	137	(128 - 153)	(107 - 161)	1	0	150.5	(135 - 155)	(119 - 167)	0	0
Platelets ($x10^9$/L)	241	(223 - 275)	(129 - 335)	1	0	219	(206 - 262)	(189 - 323)	0	0
WBC ($x10^9$/L)	5.5	(4.1 - 6.1)	(3.3 - 8.4)	4	0	6.15	(5.1 - 6.6)	(3.7 - 8.2)	1	0
Neutrophils ($x10^9$/L)	3.1	(2.6 - 4.5)	(1.4 - 6.1)	1	0	3.75	(3.05 - 4.15)	(2 - 6.5)	0	0
Lymphocytes ($x10^9$/L)	1.2	(0.9 - 1.7)	(0.5 - 2.8)	3	0	1.4	(1.2 - 1.85)	(0.8 - 2.3)	0	0
Monocytes ($x10^9$/L)	0.5	(0.4 - 0.6)	(0.4 - 0.8)	0	0	0.45	(0.4 - 0.55)	(0.3 - 0.7)	0	0
Eosinophils ($x10^9$/L)	0.1	(0.1 - 0.2)	(0 - 0.4)	0	0	0.15	(0.1 - 0.25)	(0.1 - 0.5)	0	0
Basophils ($x10^9$/L)	0	(0 - 0)	(0 - 0.1)	0	0	0	(0 - 0.1)	(0 - 0.1)	0	0
**Biochemistry**										
Bilirubin (μmol/L)	7	(6 - 9)	(4 -13)	0	0	7	(6 - 9)	(4 - 11)	0	0
ALT (U/L)	18	(14 - 22)	(10 - 37)	0	0	24.5	(17 - 29)	(12 - 44)	0	0
ALP (U/L)	60	(55 - 84)	(42 - 106)	0	0	75	(61 - 90)	(37 - 130)	0	1
AST (U/L)	20	(17 - 25)	(12 - 31)	0	0	22.5	(20.5 - 26.5)	(15 - 32)	0	0
Albumin (g/L)	38	(36 - 39)	(34 - 41)	2	0	38.5	(37 - 39.5)	(35 - 42)	0	0
Total protein (g/L)	70	(67 - 74)	(63 - 75)	1	0	70	(67 - 73)	(63 - 78)	2	0
GGT (U/L)	20	(16 - 24)	(7 - 54)	0	0	22.5	(19.5 - 32.5)	(14 - 135)	0	1
Urea (mmol/L)	5.5	(4.5 - 6.1)	(3.1 - 7.2)	1	0	4.75	(4.3 - 6.1)	(2.8 - 8.6)	1	1
Sodium (mmol/L)	140	(138 - 141)	(133 - 142)	1	0	141	(139.5 - 142)	(137 - 146)	0	1
Potassium (mmol/L)	4.3	(4.2 - 4.5)	(3.6 - 4.8)	0	0	4.35	(4.2 -4.4)	(4 - 4.7)	0	0
LDH (U/L)	398	(337 -417)	(296 - 537)	0	1	377.5	(324.5 - 411)	(246 - 476)	0	0
Creatinine (μmol/L)	77	(65 - 68)	(57 - 92)	0	0	79	(62 - 90.5)	(53 - 112)	0	1
**Coagulation**										
APTT (sec)	26	(24 - 27)	(22 - 31)	3	0	25	(24 - 26.5)	(23 - 29)	1	0
Fibrinogen assay (g/L)	2.9	(2.4 - 3.4)	(2.1 - 3.9)	0	0	3.05	(2.75 - 3.45)	(2.5 - 5.1)	0	1
INR (ratio)	1	(1 - 1)	(1 - 1.3)	0	1	1	(1 - 1.1)	(0.9 - 1.3)	0	1
Prothrombin time (sec)	12.2	(12 - 12.5)	(11.6 - 15.2)	0	1	12.1	(11.7 - 12.55)	(11.2 - 15.2)	0	1

Supplementary Table 2 Antibody panels used for flow cytometry. Marker, fluorophore, clone, identifier, source and dilution are indicated.

Marker	Fluorophore	Identifier, RRID	Source	µL/ sample
Analysis of thawed DC vaccines:				
CD209 (DC-SIGN)	BV421	Clone 9E9A8, AB_2734324	BioLegend	0.5
CD86	BV510	Clone IT2.2, AB_2562064	BioLegend	0.5
HLA-DR	BV570	Clone L243, AB_2650882	BioLegend	2
CD274 (PD-L1)	BV650	Clone 29E.2A3, AB_2629614	BioLegend	0.5
CD83	BV711	Clone HB15e, AB_2650749	BioLegend	1
CD14	BV750	Clone 63D3, AB_2801010	BioLegend	0.25
CD80	BV786	Clone 2D10, AB_2734272	BioLegend	1
CD40	FITC	Clone HB14, AB_314967	BioLegend	1
CD25	PE	Clone BV96, AB_314276	BioLegend	0.5
CD197 (CCR7)	PE/Dazzle594	Clone G043H7, AB_2563641	BioLegend	2
CD1a	PerCP eFlour 710	Clone HI149, AB_11218089	BioLegend	0.5
CD1d	Pe-Cy7	Clone 51.1, AB_2562408	BioLegend	1
CD54	AF647	Clone HA58, AB_2715942	BioLegend	0.5
HLA-A,B,C	AF700	Clone W6/32, AB_2566306	BioLegend	2
Viability	Zombie NIR		BioLegend	1:2000 dilution
Intracellular cytokine staining (ICS) of T cells:				
CD3	PacificBlue	Clone SK7 , AB_2563421	BioLegend	1
CD4	BV510	Clone OKT4, AB_317443	BioLegend	1
CD8	BV711	Clone RPA-T8, AB_11218793	BioLegend	1
IFN-γ	PE-Cy7	Clone B27, AB_2123322	BioLegend	0.05
TNF-α	BV650	Clone MAb11, AB_2561355	BioLegend	0.5
IL-2	AF647	Clone MQ1-17H12, AB_11147166	BioLegend	0.5
Viability	Live dead fixable blue		Thermofisher	1:1000 dilution
Ex vivo NKT cell analysis:				
CD3	PacificBlue	Clone SK7 , AB_2563421	BioLegend	1
CD4	BV510	Clone OKT4, AB_317443	BioLegend	1
CD8	BV711	Clone RPA-T8, AB_11218793	BioLegend	1
α-GalCer-human CD1d tetramer	APC		ProImmune	0.2
Viability	Live dead fixable blue		Thermofisher	1:1000 dilution

Supplementary Table 3 Cytokine levels in serum in Stage I[a].

Cytokine	DCV			DCV+α-GalCer		
	% zeros[b]	Median	IQR	% zeros[a]	Median	IQR
IL-4	0	31.5	(23.8 - 36.8)	0	32.5	(25 - 39)
IL-6	3.2	7	(3.5 - 11.5)	4.4	7.5	(4.3 - 14)
IL-10	17.7	5.5	(1.3 - 11)	3.1	7.5	(3.5 - 17)
IL-12p70	6.4	17	(8.3 - 32)	0	17.3	(9.3 - 29.3)
TNF	0.3	15	(12.5 - 18)	1.3	15.8	(12 - 19)
IFN-γ	0	8.5	(6.5 - 10.5)	1.8	9.5	(6.5 - 12)
MCP-1	24.6	12	(0.3 - 23)	27.8	14	(0 - 27)
MIP-1α	0	12	(9.5 - 17)	4.4	13.5	(9.5 - 17)
MIP-1β	0	678	(457 - 895)	0	735	(576 - 1162)
RANTES	0	18811	(17851 - 19774)	0	19073	(17011 - 20800)
IP-10	0	847	(546 - 1488)	0	735	(569 - 1186)

[a] Expressed as binding of cytokine-specific antibody-coupled fluorescent beads (MFI), minus background fluorescence.

[b] Negative values, where a cytokine's fluorescence level (MFI) was lower than the background, were set to zero.

Supplementary Table 4 Adverse events post randomisation to end of Stage I.

AE Category and event (n)	DCV		DCV+α-GalCer	
	CTCAE grade		CTCAE grade	
	1	2	1	2
Blood and lymphatic system disorders				
Anaemia	1	-	-	-
Cardiac disorders				
Atrial flutter	-	-	1	-
Eye disorders				
Conjunctivitis	-	-	-	1
Gastrointestinal disorders				
Diarrhoea	1	-	-	1
Nausea	1	-	1	-
Vomiting	-	-	-	1
General disorders and administration site conditions				
Fatigue	5	-	5	-
Fever	-	-	2	-
Flu-like symptoms	1	-	2	-
Infections and infestations				
Upper respiratory infection	-	1	-	1
Musculoskeletal and connective tissue disorders				
Arthralgia	-	-	1	-
Musculoskeletal and connective tissue disorder - other	-	-	-	1
Myalgia	1	-	-	-
Pain in extremity	1	-	1	-
Neoplasms benign, malignant and unspecified (incl. cysts and polyps)	-	1	-	1
Nervous system disorders				
Dysesthesia	1	-	-	-
Dysgeusia	1	-	-	-
Headache	2	1	5	-
Nervous system disorders	1	-	-	-
Paresthesia	1	-	1	-
Respiratory, thoracic and mediastinal disorders				
Bronchial obstruction	-	-	-	1
Cough	1	-	1	-
Hoarseness	-	-	1	-
Nasal congestion	1	-	1	-
Productive cough	1	-	-	-
Sneezing	1	-	-	-
Sore throat	2	-	1	1
Skin and subcutaneous tissue disorders				
Skin and subcutaneous tissue disorders - other	1	1	1	-
Total	24	4	24	8

Supplementary Table 5 Comparison of cytokine levels for Arm 1 cross-over from DCV for cycles 1 and 2 at Stage I to DCV+α-GalCer for cycles 3 and 4 at Stage II.

Cytokine	Difference in AUC	95 % confidence interval	*P*-value
IL-4	5.91	(-1.54 to 13.36)	0.1193
IL-6	11.61	(-4.46 to 27.67)	0.1557
IL-10	12.29	(-8.32 to 32.90)	0.2404
IL-12p70	7.93	(-11.36 to 27.21)	0.4174
TNF	5.13	(-1.29 to 11.56)	0.1165
IFN-γ	3.54	(-3.90 to 10.98)	0.3495
MCP-1	10.56	(-11.59 to 32.70)	0.348
MIP-1α	10.85	(-1.26 to 22.95)	0.0787
MIP-1β	6.91	(-5.34 to 19.17)	0.2668
RANTES	7.07	(0.43 to 13.72)	0.0371*
IP-10	3.01	(-11.86 to 17.88)	0.6897

Supplementary Table 6 Adverse events at Stage II.

AE Category and event (n)	Observation		DCV+α-GalCer	
	CTCAE grade		CTCAE grade	
	1	2	1	2
Gastrointestinal disorders				
Nausea	-	-	2	-
General disorders and administration site conditions				
Fatigue	-	-	2	-
Fever	-	-	1	-
Flu like symptoms	1	-	1	-
Infections and infestations - other	1	-	-	1
Metabolism and nutrition disorders				
Anorexia	-	-	-	1
Musculoskeletal and connective tissue disorders				
Arthralgia	1	-	1	-
Myalgia	1	-	-	-
Neoplasms benign, malignant and unspecified (incl. cysts and polyps) - other	-	-	-	1
Nervous system disorders				
Headache	1	-	-	-
Respiratory, thoracic and mediastinal disorders				
Cough	-	-	2	-
Nasal congestion	-	-	1	-
Respiratory, thoracic and mediastinal disorders - other	-	-	-	1
Sore throat	-	-	1	-
Wheezing	-	-	1	-
Skin and subcutaneous tissue disorders - other	1	-	-	1
Total	6	0	12	5
